# Surgical Implications of Coronary Arterial Anatomy in Adults with Congenital Cardiac Disease

**DOI:** 10.2174/1874192400802010049

**Published:** 2008-07-16

**Authors:** Ad J.J.C Bogers, Ismael Eralp, A. Pieter Kappetein

**Affiliations:** 1 Department of Cardiothoracic Surgery, Thoraxcentre, Erasmus MC, PO Box 2040, 3000 CA Rotterdam, The Netherlands

## Abstract

In adults with congenital heart disease coronary arterial anatomy, normal as well as anomalous, may have implications in surgical reconstruction of an underlying cardiac structure.

In addition to the diagnostic imaging, necessary in surgery for adult congenital heart disease, additional information with regard to the spatial relation between the relevant cardiac structure and the coronary arterial system may be required for planning the operation and providing a good outcome.

The congenital cardiac surgeon should have the necessary skills in coronary artery bypass techniques.

With lack of adequate data, the estimation of mortality due to complications as a result of coronary damage in surgery for adult congenital cardiac disease of below 1% seems fair.

## INTRODUCTION

In adults with congenital heart disease, the origin and course of the coronary arteries, both normal as well as anomalous, may have implications in surgical reconstruction of an underlying cardiac structure. In isolated congenital coronary arterial anomalies, a wide variety in anatomy as well as pathophysiology and clinical presentation is found [1,2]. Recently this was exemplified in a review on anomalous connection of a coronary artery to the opposite sinus of the aorta, a subgroup of anomalies frequently showing intussusception of the ectopic coronary artery in between the aorta and the pulmonary artery [3].

In addition to the diagnostic imaging, necessary in surgery for adult congenital heart disease, additional information with regard to the spatial relation between the relevant cardiac structure and the coronary arterial system may be required for planning the operation and providing a good outcome.

The goal of this presentation is to increase the awareness for the spatial relationship between the coronary arterial system, either normal or anomalous, and underlying structural congenital heart disease and the possible consequences for surgery and outcome in this regard.

## RECENT FINDINGS IN CORONARY EMBRYOLOGY

Embryological studies have shown the crucial role of the pro-epicardial organ (PEO) in the development the coronary arterial system [4]. Epicardium Derived Cells (EPDC’s) from this PEO migrate to the endocardial cushions, the myocardium and subepicardium, the fibrous skeleton and impor

tantly also to the coronary vasculature [5]. It has been shown that in a process of epithelial mesenchymal transformation, the EPDC’s differentiate into myocardial, subepicardial and perivascular fibroblasts and coronary arterial smooth muscle cells [6,7]. The EPDC’s are essential for myocardial maturation, myocardial vascularization, stimulation of coronary vascular formation, building up the medial layer of the coronary arteries and for ingrowth of the coronary arteries into the great arteries, usually the aorta [8]. All these studies confirmed that coronary arterial development concentrates in three identifiable rings, the peritruncal ring, containing the coronary arterial orifices, the atrioventricular groove ring and the interventricular septal ring [9].

## CORONARY ARTERIAL ANOMALIES

With regard to the results of the embryological studies, it is not surprising that the vast majority of coronary arterial anomalies are found in the area of the three rings.

Among the anomalies associated with myocardial ischemia are coronary fistula’s, the abnormal right or left coronary artery connected to the pulmonary artery (ARCAPA or ALCAPA), congenital coronary stenosis or atresia, anomalous origin from the contralateral sinus [3] and a single orifice with a branch running between the aorta and pulmonary artery [1-3].

Anomalies coronary arteries usually not associated with myocardial ischemia are a left circumflex coronary artery originating from the right sinus and the coronary arteries originating from one sinus with separate orifices [1-3].

The incidence, clinical characteristics and possible therapeutical interventions for these coronary arterial anomalies are well described. Nevertheless, attempts at further classification of coronary anomalies are being produced, a recent contribution being made with regard to the anomalous origin of a coronary artery from the opposite sinus [3].

All of these can be regarded as isolated anomalies [1,2]. Exact figures on their prevalence are not available, presented numbers being dependant on differences in definition, sampling and background cohort [3]. It seems reasonable to assume that the figure in patients is below 1% and in the general population probably below 0.01% [1-3].

## TREATMENT OPTIONS IN ISOLATED CONGENITAL ANOMALIES OF PROXIMAL AORTIC CORONARY ARTERIES

Not all cases of isolated congenital anomalies of the coronary arteries warrant treatment [1,3]. For instance the right abnormal coronary artery connected to the opposite sinus much less frequently needs treatment as compared to the left coronary artery connected to the opposite sinus [3]. In symptomatic patients, the treatment options in isolated anomalies of the proximal coronary arteries connected to the aorta are guided by clinical presentation. Essentially 3 treatment options are relevant: medical or observative treatment, percutaneous intervention with stent deployment or surgical treatment [3].

Surgical options should depend on the local anatomy and the surgical expertise and may consist of reimplantion of the ectopic coronary arterial orifice, unroofing of the intramural coronary segment or creating of an additional aortic orifice at the end of the intramural course of the coronary artery [1,3]

## SURGICAL RELEVANCE

In adults with congenital heart disease, the origin and course of coronary arteries have implications in surgical reconstruction of an underlying cardiac structure, The spatial relation between the relevant cardiac structure and the coronary arterial system is essential for planning the operation and providing a good outcome. Three-dimensional representation of the diagnostic data plays an increasingly important role in this regard.

It is important to realize that not only the coronary anomalies pose a risk in cardiac surgery in adults with congenital heart disease, but also injury of the normal coronary artery system.

The diagnostic sequence may therefore start with the usual echocardiography [3]. This will provide spatial information on the structural abnormalities and may provide data on the proximal coronary arterial anatomy as well. If however this information is inconclusive, computed tomography or magnetic imaging is recommend, directed at both cardiac as well as coronary anatomy and their interrelationship [3]. Coronary angiography is important to document the proximal and further course as well as the luminal quality of the coronary arteries, but is less optimal to provide anatomical data on the interrelationship with cardiac structures, even with contemporary contrast injection of cardiac cavities. It is of utmost importance that a final inspection is performed at surgery before irreversible surgical steps are being made.

## SURGERY NEAR THE PROXIMAL CORONARY ARTERIES

The proximal parts of both normal and abnormal coronary arteries are at risk in surgery near the proximal coronary arteries. In any aortotomy the surgeon should be aware of the position of the coronary orifices and the course of the proximal segment of the coronary arteries. Harvesting the pulmonary root in an autograft procedure may damage the left main coronary artery or the first septal branch. In any primary or redo surgical procedure of the right ventricular outflow tract (e.g. tetralogy of Fallot, pulmonary atresia, common arterial trunk) a coronary arterial branch may also be at risk for injury. This may include a left anterior descending coronary artery (LAD) from the right coronary sinus, a single right coronary artery (RCA) with an LAD anterior to the pulmonary trunk, a single left coronary artery with an RCA anterior to the pulmonary trunk or a large infundibular branch from the RCA. In pulmonary root surgery (e.g. allograft implantation) the left main coronary artery at the posterior annular level of the pulmonary orifice may also be at risk. In most of these patients atherosclerotic coronary disease does not play a role, probably because of the younger age at operation 

No hard data are available on actual damage, but it seems reasonable to estimate mortality due to complications as a result of coronary arterial damage up to 1%.

## SURGERY ON THE CORONARY ORIFICES

Also the coronary arterial orifices themselves may be at risk for damage in aortic root surgery. Particularly when coronary orifices are replanted in root replacement surgery as in the autograft procedure or during prosthetic valve composite graft root replacement. Especially when the coronary buttons have to be re-excised and re-implanted again, scar tissue and local fibrosis and calcification may pose an additional challenge in the pursuit of successful surgery. In case of surgery in common arterial trunk, the increased variability of the position of the coronary arterial orifices should be adequately appreciated [10]. Despite the fact that coronary arterial anatomy is not any longer considered a risk factor for adverse outcome in the neonatal arterial switch operation, this may not necessarily be the case in the double switch procedure. Adult coronary orifices and proximal coronary arteries are definitely less pliable than in children. In addition, the incidence of anomalies of the proximal coronary arteries in corrected transposition of the great arteries is increased. Because of the young age of the patients atherosclerotic orifice degeneration does not play a role at this moment but may play a role in the future.

Although no firm data are available, again it seems fair to estimate mortality due to complications as a result of coronary damage in this regard at up to 1%.

## CORONARY ARTERIES AS MEANS OF TRANSPORT

In all surgical procedures that involve opening of the aortic root, the coronary orifices are at risk of damage due to manipulation. The risk is small, but direct application of cardioplegia into the orifices may be traumatic. In addition there is the risk of coronary embolism by gas (air or carbon dioxide) or debris after completion of the root surgery.

## CONCLUSIONS

Not only abnormal, but also normal coronary arterial anatomy may have implications in surgery for adult congenital heart disease.

Three-dimensional diagnostic tools demonstrating the spatial relation between coronary arteries and the cardiac region of interest are essential.

The congenital cardiac surgeon should have the necessary skills in coronary artery bypass techniques.

With lack of adequate data, the estimation of mortality due to complications as a result of coronary damage in surgery for adult congenital cardiac disease of below 1% seems fair.

So far, clinical atherosclerotic coronary disease in adults with congenital cardiac disease is infrequent.
